# Full-waveform acoustic tomography for fluid temperature and flow

**DOI:** 10.1007/s00348-025-04068-z

**Published:** 2025-07-08

**Authors:** Lennart Kira, Jerome Noir

**Affiliations:** Department of Earth and Planetary Sciences, ETH Zürich, Sonneggstrasse 5, 8090 Zurich, Switzerland

## Abstract

**Abstract:**

Using the travel time of sound waves advected by a moving carrier medium, acoustic tomography allows to reconstruct temperature and flow fields in opaque fluids without tracers or scattering particles. Reconstruction algorithms are conventionally based on the ray approximation and pose difficulties, especially in enclosed domains: Interferences of early reflections can prevent the assignment of each arrival to the correct ray path. We develop a full-waveform inversion for acoustic tomography in laboratory-scale experiments, perform synthetic tests, and benchmark these with a straight-ray algorithm. Multiple late arrivals of reflected waves are considered in order to increase the quality of the reconstructions when restricted to a sparse transducer array. In addition, the full-waveform algorithm allows to invert simultaneously emitted signals from all sources, decreasing the acquisition time in which a flow must be assumed stationary. These findings make the new method especially interesting for researchers experimenting with enclosed, opaque fluids where no optical imaging is feasible. Furthermore, we envision a potential application of the newly developed method to map flows around objects or complex wall geometries and even multiphase flows.

**Graphical abstract:**

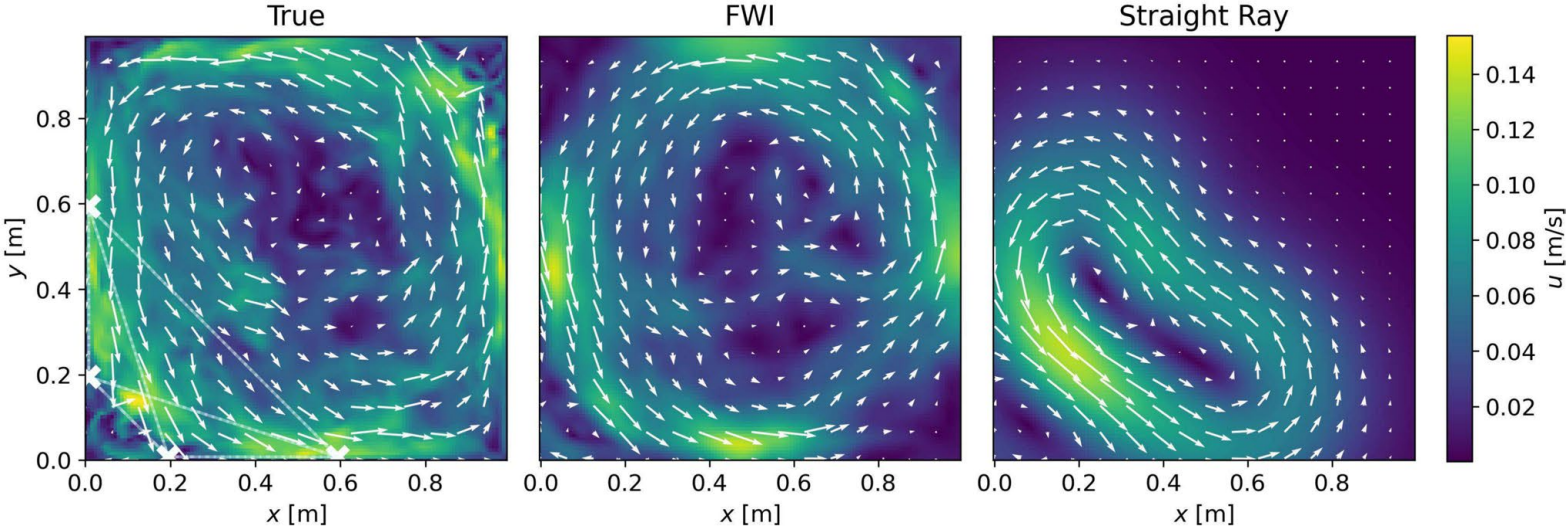

**Supplementary Information:**

The online version contains supplementary material available at 10.1007/s00348-025-04068-z.

## Introduction

The time of flight (ToF) method is a technique for measuring flow velocities in fluids or gases by observing travel times of advected sound waves (Kalmus 1954). Ultrasonic ToF has found use, e.g., in medical blood flow measurements (Franklin et al. 1959), in wind speed measurements (Schotland 1955) or for monitoring pipe flows (a review on ToF flow meters is provided by Lynnworth and Liu (2006)). The ToF method is nonintrusive as the sensors may be installed outside of the domain of interest and, unlike imaging methods, such as particle imaging velocimetry (PIV) or particle tracking velocimetry (PTV), it may be used in opaque fluids. Measurements furthermore do not rely on reflecting particles inside the fluid, as needed for ultrasonic Doppler velocimetry or laser Doppler velocimetry.

Using the ray formulation, the travel time of a sound wave traveling from an emitter at point ${\varvec{x}}_a$ to a receiver at point ${\varvec{x}}_b$ is determined as

1 $$\begin{aligned} \tau _{ab} = \int _{{\varvec{x}}_a}^{{\varvec{x}}_b} \frac{dl}{c_\text {eff}}, \end{aligned}$$

Where the *effective* sound speed is defined by

2 $$\begin{aligned} c_\text {eff} = c({\varvec{x}}, t) + {\varvec{u}}({\varvec{x}}, t) \cdot \hat{{\varvec{k}}}, \end{aligned}$$

with the acoustic sound speed of the medium $c({\varvec{x}}, t)$, the fluid velocity ${\varvec{u}}({\varvec{x}}, t)$ and the unit ray vector $\hat{{\varvec{k}}}$, perpendicular to the wave fronts pointing in the direction of travel (e.g., Barth and Raabe (2011)). Furthermore, when assuming $|{\varvec{u}}|<< c$, Eq. (1) can be recast as

3 $$\begin{aligned} \tau _{ab} \approx \int _{{\varvec{x}}_a}^{{\varvec{x}}_b}\frac{dl}{c} - \int _{{\varvec{x}}_a}^{{\varvec{x}}_b}\frac{{\varvec{u}} \cdot \hat{{\varvec{k}}}\, dl}{c^2}. \end{aligned}$$

Making use of $\hat{{\varvec{k}}}$ inverting its sign on the reciprocal path from ${\varvec{x}}_b$ to ${\varvec{x}}_a$, it is a common technique to use either the sum

4 $$\begin{aligned} \tau _{ba} + \tau _{ab} = 2\,\int _{{\varvec{x}}_a}^{{\varvec{x}}_b}\frac{dl}{c} \end{aligned}$$

or the difference

5 $$\begin{aligned} \tau _{ba} - \tau _{ab} = 2\, \int _{{\varvec{x}}_a}^{{\varvec{x}}_b}\frac{{\varvec{u}} \cdot \hat{{\varvec{k}}}\, dl}{c^2}, \end{aligned}$$

of the reciprocal travel times $\tau _{ba}$ and $\tau _{ab}$ to separate the effects of sound speed and flow. This allows to invert only for the sound speed using Eq. (4) before inverting for the flow using Eq. (5) (Norton 1989). Assuming a homogeneous fluid, a sound speed anomaly can only be introduced by a temperature anomaly $\Delta T$ along the path. Thus, having obtained the sound speed, the temperature may simply be extracted by inverting the relation $c(\Delta T)$.

Many tomographic algorithms have been proposed to estimate two- or three-dimensional flow fields by inverting travel time data of multiple source–receiver pairs. Acoustic tomography (ATOM) is a method of increasing popularity (Othmani et al. 2023). It provides a means to obtain these fields without the need for tracer particles or optical transparency, making it a promising substitution for the PIV and PTV methods in opaque fluids.

To the best of our knowledge, ATOM has so far always been applied using the ray approximation (see, e.g., Ostashev et al. (2008), Othmani et al. (2023) and references therein). The inversion consists of finding an optimal model of *c* and ${\varvec{u}}$ such that the travel times $\tau$ predicted by this synthetic model have a minimal misfit with the true, observed travel times. The reader is referred to Othmani et al. (2023) for an overview on the most popular inversion algorithms in ATOM.

### Limitations of the ray approximation

Most proposed ATOM schemes only evaluate first arrivals and assume straight, direct rays between the source and receiver to keep the forward problem, i.e., the estimation of travel times, simple. This omits information carried by reflections if the experiment is conducted in an enclosed space. Bleisteiner et al. (2016) show that by using only one omnidirectional source–receiver pair, a large room can be probed by a substantial number of rays to obtain a three-dimensional temperature field. In enclosed domains, the additional ray coverage granted by boundary-reflected waves can thus tackle the common challenge of under-determinedness faced in ATOM (Wilson and Thomson 1994). Using the ToF method with single rays, Burmann et al. (2022) demonstrate that reflected sound rays, due to their longer ray paths, provide a significantly better signal to noise ratio than direct rays when reconstructing zonal flows in a rotating cylinder. To the best of our knowledge, no ATOM scheme has been proposed using reciprocal travel times of reflected signals to infer flow velocities in enclosed domains (Othmani et al. 2023).

The need to assign a ray path to each measured arrival time is a major challenge when early reflections are taken into account in a tomographic inversion. A scheme for optimal positioning of one source–receiver pair, maximally separating the arrival times, has been proposed (Dokhanchi et al. 2020) but might not guarantee success for several transducer pairs or smaller experimental domains.

Another challenge of reconstructing flow fields is that the flow patterns may change over time. Hence, a strict requirement for using the ray approximation is that multiple signals must be distinguishable if sound is emitted simultaneously from all sources. The alternative is releasing signals consecutively in time (e.g., Johnson et al. (1977), Gan et al. (2003), Barth et al. (2007), Barth and Raabe (2011)) which drastically reduces temporal resolution and might allow the flow field to change significantly throughout the acquisition. Wiens and Behrens (2009) or Li et al. (2011) tackle this challenge by encoding each signal of the transmitters by a distinct frequency or Kasami sequence, respectively, thus allowing all probes to record while simultaneously emitting sound. However, such approaches require transmissions lasting long enough to allow for separating the signals in the spectral domain. This could cause early reflections to leak into the first arrivals if applied in small, enclosed domains. Thus, the fact that the reconstruction relies on the correct assignment of each signal to a distinct ray path clearly becomes an impediment.

### Alternative approaches

Apart from ATOM in the ray approximation, there are two different approaches to reconstruct flow fields by evaluating acoustic waves in fluids—both deriving from helioseismology.

One of them is modal acoustic velocimetry (MAV), a method based on observing the splitting of frequency peaks of acoustic modes due to zonal flows inside a cavity. Unfortunately, it is necessary to first predict different modes to use MAV in containers of different shapes. So far MAV has only been considered for simple geometries such as spherical, spheroidal and ellipsoidal cavities (Triana et al. (2014), Su et al. (2020) and Vidal et al. (2020), respectively).

Hanasoge et al. (2011) propose a full-waveform inversion (FWI) in helioseismology. FWI is primarily used by seismologists (see Virieux and Operto (2009) for an overview) and has also been introduced to medical ultrasonic imaging (e.g., Marty et al. (2021)). It can briefly be summarized as a method which minimizes the misfit between full, observed waveforms and those predicted by a forward simulation of the full wavefield inside the domain of interest, using an estimated wave speed and flow model for arbitrary geometries. In contrast to ray-based algorithms, FWI does not require the picking of any arrivals and the assignment of these to distinct ray paths. By directly comparing the simulated and measured waveforms, one may automatically invert the entire acoustic signal for the parameters of interest, where the information carried by multiple reflections can be included into the reconstructions without much effort.

Hanasoge (2014) has synthetically tested the method’s ability to recover flow fields in the interior of the Sun and concludes that a reconstruction thereof proves more difficult than reconstructions of structural parameters using FWI in a previous study (Hanasoge and Tromp 2014), due to a slow reduction of the misfit by optimizing the flow field.

However, we will demonstrate that in laboratory-scale, enclosed domains, with the usage of controlled sources, the FWI may provide critical advantages compared to a straight-ray analysis in certain scenarios.

This article is structured in the following manner. In Sect. 2, we present the theoretical background on how we model the wave propagation as well as an introduction to the FWI. We describe the numerical discretization of the model in Sect. 3. Additionally, we present the straight-ray code used to benchmark our inversion results. The reconstructions are presented in Sect. 4, which form the basis of our discussion on the benefits and challenges associated with the FWI in Sect. 5.

## Theoretical background

### The forward model

First, we derive a differential equation describing the propagation of an advected sound wave. The inspiration to the final equation originates from the seismic wave equation in helioseismology (Hanasoge et al. 2011; Hanasoge 2014). To derive a forward model suitable for ATOM, we start with the incompressible Navier–Stokes equation for the background flow ${\varvec{u}}$:

6 $$\begin{aligned} \rho \, \left( \frac{\partial }{\partial t} + {\varvec{u}} \cdot \nabla \right) {\varvec{u}} = -\nabla p + \rho \nu \nabla ^2 {\varvec{u}} \end{aligned}$$

7 $$\begin{aligned} \nabla \cdot {\varvec{u}} = 0\,, \hspace{0.4in} \left( \frac{\partial }{\partial t} + {\varvec{u}} \cdot \nabla \right) \rho = 0 \end{aligned}$$

*p* and $\rho$ are the pressure and density of the fluid, respectively, and $\nu$ is the kinematic viscosity.

Furthermore, if an isochemical fluid is assumed, the density varies only due to variations in the temperature $T({\varvec{x}}, t)$ of the fluid.

8 $$\begin{aligned} \rho ({\varvec{x}}, t) = \rho _0 + \overline{\rho }({\varvec{x}}, t) = \rho _0\,(1 + \alpha \, \Delta T({\varvec{x}}, t)), \end{aligned}$$

where the constant background density is denoted by $\rho _0$ and the density variation by $\overline{\rho }({\varvec{x}}, t) = \rho _0 \, \alpha \, \Delta T({\varvec{x}}, t)$. $\alpha$ is the volumetric expansion coefficient and $\Delta T = T({\varvec{x}}, t) - T_0$ is an anomaly from a constant background temperature $T_0$.

As a sound wave, represented by the displacement ${\varvec{\xi }} ({\varvec{x}}, t)$, travels through the fluid, the background velocity is perturbed.

9 $$\begin{aligned} {\varvec{u}}' = {\varvec{u}} + \tilde{{\varvec{u}}}\,,\hspace{0.2cm} \text {where} \hspace{0.3cm} \tilde{{\varvec{u}}} = \left( \frac{\partial }{\partial t} + {\varvec{u}} \cdot \nabla \right) {\varvec{\xi }} \end{aligned}$$

and the tilde denotes a perturbation due to wave motion. Furthermore, the pressure and the density are perturbed as well and can be rewritten as

10 $$\begin{aligned} p' = p + \tilde{p} \hspace{0.5cm} \text {and} \hspace{0.5cm} \rho ' = \rho _0 + \overline{\rho }+ \tilde{\rho }. \end{aligned}$$

The perturbed Navier–Stokes equation can thus be written as

11 $$\begin{aligned} \rho ' \, \left( \frac{\partial }{\partial t} + {\varvec{u}}' \cdot \nabla \right) {\varvec{u}}' = -\nabla p' + \rho ' \nu \nabla ^2 {\varvec{u}}' \end{aligned}$$

12 $$\begin{aligned} \left( \frac{\partial }{\partial t} + {\varvec{u}}' \cdot \nabla \right) \rho ' = - \rho ' \,\nabla \cdot {\varvec{u}}'\,, \end{aligned}$$

Note that the continuity equation (12) now takes into account the compressibility of the perturbed flow $\tilde{{\varvec{u}}}$.

Equations (11) and (12) are nonlinear in ${\varvec{\xi }}$. However, the problem can be linearized in most applications using scale separation. This is a tedious exercise, and a detailed derivation is provided in the supplementary material (Online Resource 1) for the interested reader. However, the main assumptions can be summarized in the following manner.

Let us denote the typical sound speed by $c_0$, the wavelength by $\lambda$, the typical length scale of the temperature and velocity gradient by *L* and the typical flow speed by $u_0$. In a laboratory-scale setting involving subsonic flows, we may assume the following hierarchical scaling:

13 $$\begin{aligned} \left( \frac{|{\varvec{\xi }}|}{\lambda }, \frac{|\tilde{\rho }|}{\rho _0}, \frac{\tau _c}{\tau _\nu }\right)<< \left( \frac{\lambda }{L},\, \frac{u_0}{c_0}\right)<< 1 \hspace{0.2cm}, \end{aligned}$$

with the acoustic wave timescale $\tau _c = \lambda /c_0$ and the timescale $\tau _\nu = \lambda ^2/\nu$, representing the typical viscous diffusion time over a wavelength. This scaling is applicable for, e.g., an experiment in air and an example is provided in the supplementary material (Online Resource 1).

If we retain only the largest terms in the dynamic balance, assuming the validity of our hierarchical scaling, we arrive at the first order equation

14 $$\begin{aligned} \frac{\partial ^{2}}{{\partial t}^{2}} {\varvec{\xi }} = -\frac{\nabla \tilde{p}}{\rho }, \end{aligned}$$

in which the background flow does not appear. The flow field enters the dynamics only as a second-order term, where the approximate equation takes the form

15 $$\begin{aligned} \frac{\partial ^{2}}{{\partial t}^{2}} {\varvec{\xi }} + 2{\varvec{u}}\cdot \nabla \frac{\partial }{\partial t} {\varvec{\xi }} = -\frac{\nabla \tilde{p}}{\rho }. \end{aligned}$$

The new term accounts for the advection of the wave displacement ${\varvec{\xi }}$.

Assuming an elastic constitutive relation between the wave displacement and the perturbation pressure, we have

16 $$\begin{aligned} \tilde{p} = -\kappa \nabla \cdot {\varvec{\xi }} = -\rho c^2 \nabla \cdot {\varvec{\xi }}, \end{aligned}$$

where $\kappa = \rho \, c^2$ is the isentropic bulk modulus of the fluid.

Substituting this into the divergence of the wave Eq. (15), we obtain

17 $$\begin{aligned} \mathcal {L}p = \left[ \frac{1}{c^2}\, \left( \frac{\partial ^{2}}{{\partial t}^{2}} + 2{\varvec{u}}\cdot \nabla \frac{\partial }{\partial t} \right) - \nabla ^2 \right] p = 0, \end{aligned}$$

where the linear propagation operator $\mathcal {L}$ depends on the sound speed and velocity in the domain. The tilde, indicating the perturbation pressure, has been omitted for clarity; thereafter, *p* will refer to the perturbation pressure unless otherwise indicated.

In the present study, we consider the case of a bounded domain *V*, for which a non-penetration condition

$\hat{{\varvec{n}}} \cdot {\varvec{\xi }}({\varvec{x}}, t) = 0$ must be satisfied at the boundary $\partial V$. By multiplying Eq. (15) with the outer normal vector $\hat{{\varvec{n}}}$ of the domain, a Neumann boundary condition for the pressure can be written as

18 $$\begin{aligned} \hat{{\varvec{n}}} \cdot \nabla p({\varvec{x}}, t) = 0 \hspace{0.1cm}, \hspace{1cm} {\varvec{x}} \in \partial V. \end{aligned}$$

### The adjoint method in FWI

The objective of the FWI is to recover the sound speed and velocity fields (c, ${\varvec{u}}$) that provide the best match between the observed ($p^\text {obs}(t)$) and predicted (*p*(*t*)) waveforms at each receiver. We shall now outline the fundamental principle of this inversion technique. Let us introduce the model parameters

19 $$\begin{aligned} {\varvec{m}} = \begin{bmatrix} c(\Delta T) \\ u_{x} \\ u_{y} \end{bmatrix} = (c, {\varvec{u}}). \end{aligned}$$

The quantity of interest in ATOM is conventionally the temperature anomaly $\Delta T$ which can be easily obtained by the inverse of the relation $c(\Delta T)$. For instance, in the case of an ideal gas this material equation reads

20 $$\begin{aligned} c(\Delta T) = c_0 \sqrt{1 + \frac{\Delta T}{T_0}}. \end{aligned}$$

To evaluate the correctness of an estimated model ${\varvec{m}} = (c, {\varvec{u}})$, a misfit functional must be defined that quantitatively provides a measure of the residual between the observed waveforms and the waveforms predicted by a forward simulation using the differential operator $\mathcal {L}$ in Eq. (17). In this study, we use an $L_2$-misfit between the observed and the predicted pressure waveforms at all given receiver positions ${\varvec{x}} = {\varvec{x}}_r$, which is defined as follows.

21 $$\begin{aligned} \chi = \int \displaylimits _V \int \displaylimits _{\left[ 0, \mathcal {T}\right] } \frac{1}{2}\,\left[ p({\varvec{x}}, t) - p^\text {obs}(t)\right] ^2\delta ({\varvec{x}} - {\varvec{x}}_r) \,\textrm{d}t\,\textrm{d}V \end{aligned}$$

The integral is taken over all positions ${\varvec{x}}$ in the domain *V* and all times *t* in the interval $\left[ 0, \mathcal {T}\right]$, where $\mathcal {T}$ is the duration of the measured signal.

Starting from an initial model ${\varvec{m}}$, we ought to estimate the model update $\delta {\varvec{m}}$ that reduces the misfit value. Here, we adopt the so-called adjoint method (see Fichtner (2010) Ch. 8 for a detailed derivation of the following concepts).

Let us introduce the adjoint operator $\mathcal {L}^\dag$ and the adjoint pressure field $p^\dag$, which satisfy

22 $$\begin{aligned} \langle \mathcal {L} p, p^\dag \rangle = \langle p, \mathcal {L}^\dag p^\dag \rangle , \end{aligned}$$

where the inner product of two space- and time-dependent functions *a* and *b* is defined by

23 $$\begin{aligned} \langle a, b \rangle = \int \displaylimits _V \int \displaylimits _{\left[ 0, \mathcal {T}\right] } a({\varvec{x}}, t) \, b({\varvec{x}}, t) \textrm{d}t\,\textrm{d}V. \end{aligned}$$

We furthermore introduce the sensitivity kernels

24 $$\begin{aligned} {\varvec{K}}({\varvec{x}}) = \begin{bmatrix} K_c({\varvec{x}}) \\ K_{u_{x}}({\varvec{x}}) \\ K_{u_{y}}({\varvec{x}}) \end{bmatrix} = \int _T p^\dag \, \left[ \nabla _m \mathcal {L}\, p \right] \textrm{d}t, \end{aligned}$$

where $\nabla _m \mathcal {L}$ is the gradient of $\mathcal {L}$ with respect to the three model parameters *c*, $u_x$ and $u_y$—which is known from Eq. (17).

These sensitivity kernels ${\varvec{K}}({\varvec{x}})$ are the key quantities in the FWI, since they relate a given variation $\delta {\varvec{m}}$ of the model parameters to a variation $\delta \chi$ of the misfit functional by

25 $$\begin{aligned} \delta \chi = \int _V {\varvec{K}}({\varvec{x}}) \cdot \delta {\varvec{m}}({\varvec{x}}) \, \textrm{d}V\,\,. \end{aligned}$$

For our choice of an $L_2$-misfit, the specific adjoint wavefield required for (24) can be obtained by solving the following *adjoint equation* (Fichtner 2010).

26 $$\begin{aligned} \mathcal {L}^\dag p^\dag = (p^\text {obs} - p)\delta ({\varvec{x}} - {\varvec{x}}_r) \end{aligned}$$

To solve this equation, we first need to derive the adjoint operator $\mathcal L^\dag$, which is unknown at this point.

To do so we will assume that the adjoint wavefield is subject to the same scale separation from the background quantities as the forward wavefield, denoted by the hierarchical scaling (13).

Furthermore, as classically done in FWI, we will assume the zero initial condition

27 $$\begin{aligned} p({\varvec{x}}, 0) = \frac{\partial }{\partial t} p({\varvec{x}}, 0) = 0. \end{aligned}$$

for the forward pressure field, that is, we assume no wave motion before the activation of any source. Another canonical constraint for the adjoint wavefield is the terminal condition

28 $$\begin{aligned} p^\dag ({\varvec{x}}, \mathcal {T}) = \frac{\partial }{\partial t} p^\dag ({\varvec{x}}, \mathcal {T}) = 0. \end{aligned}$$

Finally, we impose the same boundary condition for $p^\dag$ as for *p*,

29 $$\begin{aligned} \hat{{\varvec{n}}} \cdot \nabla p^\dag ({\varvec{x}}, t) = 0 \hspace{0.1cm} \text {on} \hspace{0.1cm} \partial V. \end{aligned}$$

Using the above stated assumptions, it can be shown that

30 $$\begin{aligned} \mathcal {L}^\dag = \mathcal {L}\, . \end{aligned}$$

That is, the forward operator is self-adjoint and the adjoint wavefield is governed by the same dynamics as the forward wavefield. For a detailed derivation of the adjoint operator, see the supplementary material (Online Resource 1).

To set this result into physical context, the adjoint equation can be interpreted as a wave equation with a source term $f^\dag = (p^\text {obs} - p)\delta ({\varvec{x}} - {\varvec{x}}_r)$. The adjoint wavefield is thus formed by back-propagating the errors between observed and estimated waveforms, injected at the receiver positions ${\varvec{x}}_r$, and the sensitivity kernels are a function of this error-related wavefield.

**Fig. 1:**
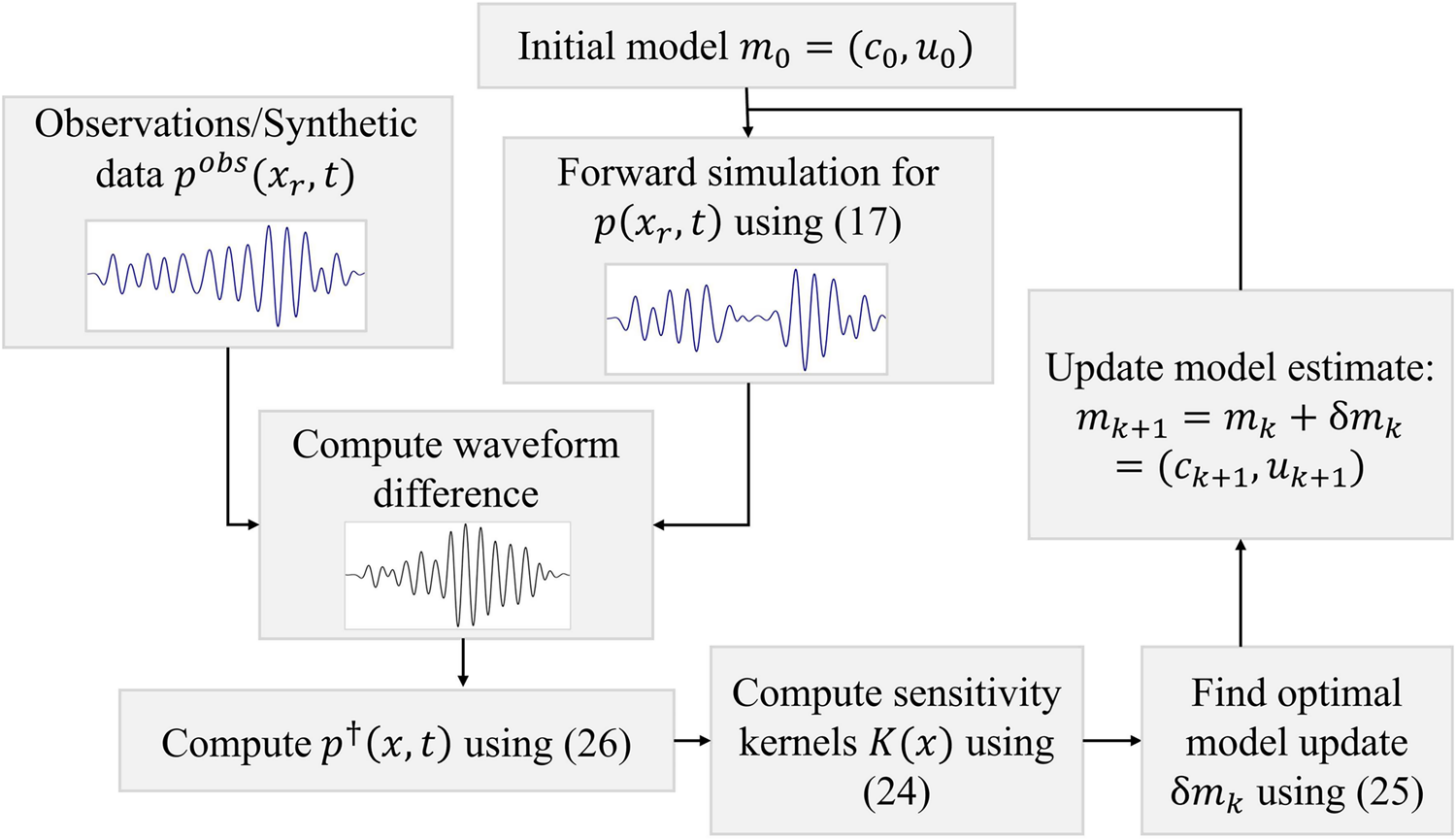
Flowchart summarizing the steps involved in the FWI using the adjoint method

To summarize the concepts above, a workflow for an iterative inversion using the adjoint method is presented in Fig. 1.

Using an initial best guess ${\varvec{m}}_0$ of the model, we propagate a forward pressure field using (17) to obtain estimated waveforms at the receiver positions ${\varvec{x}}_r$. Then, we calculate the difference between the observed and estimated waveforms, which we use as a source term for the adjoint Eq. (30). Having solved this equation for $p^\dag$, we can form the sensitivity kernels through Eq. (24). The next step is to find an optimal model update $\delta {\varvec{m}}$ given ${\varvec{K}}$. The specific optimization strategy depends on the numerical scheme and is outlined in Sect. 3.4. New estimated waveforms are then generated with the updated model and this process is iterated until the misfit is sufficiently reduced.

## Numerical methods

In this study, we aim at testing the general feasibility of the FWI by generating the observed waveforms using the forward propagation model (17) and synthetic temperature and velocity fields. The numerical implementation is based on the finite difference forward solver of the *Pestoseis* library for the acoustic wave Eq., which we have modified to account for the second-order advection term appearing in (17).

For all simulations, we make use of the *Python* library *PyTorch* and its capability to compute on a graphics processing unit (GPU). All calculations are carried out on a desktop computer with a *NVIDIA-GeForce RTX 4090*.

### Synthetic temperature and flow data

For all our simulations, we assume a square model domain filled with air, where the typical sound speed is $c_{0} = 346\frac{{\text{m}}}{{\text{s}}}$ in the absence of any temperature anomaly ($T_0 = 300\,$K).

We perform inversions for three different background fields $(\Delta T,{\varvec{u}})$ obtained from numerical simulations.

**Fig. 2:**
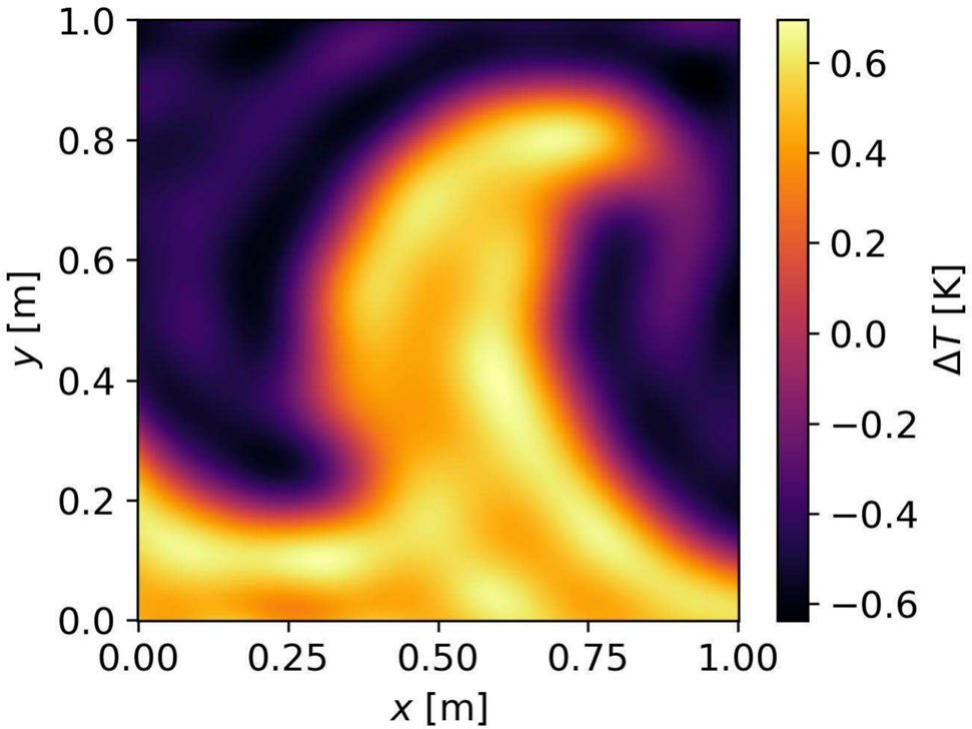
Temperature anomaly $\Delta T_\text {true}({\varvec{x}})$ of the plume-model provided by Wiens and Behrens (2009)

We start by considering a temperature anomaly in the absence of any flow ($\Delta T \ne 0, {\varvec{u}} = 0$). Any realistic numerical simulations with nontrivial temperature gradients requires nonzero flows. However, to assess the accuracy of an independent temperature reconstruction, we use data of a numerical simulation and treat the model as a stationary temperature anomaly, enclosed in a square domain by setting the flow ${\varvec{u}}$ of the original data to zero and enforcing the condition (18) at the boundary of the domain of interest. The temperature field we choose is created by a Von Kármán Vortex downstream of the flow past a cylinder, which has been used by Wiens and Behrens (2009) to assess their own ATOM based on a straight-ray approximation. It is shown in Fig. 2.

Given the sharp contours of the plume, we consider this anomaly well-suited for assessing the capabilities of our method.

**Fig. 3:**
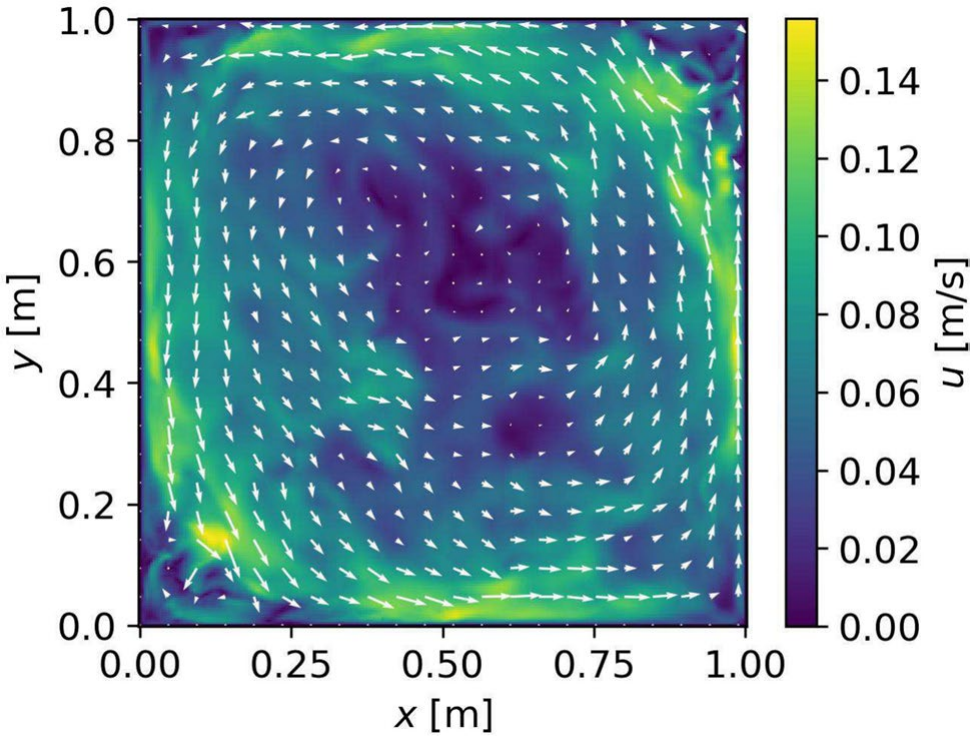
Snapshot of the flow field ${\varvec{u}}_\text {true}({\varvec{x}})$ inside a precessing cylinder provided by Giesecke et al. (2024). The Cartesian coordinate *y* corresponds to the cylindrical coordinate *z*. Colors show the absolute flow speed $|{\varvec{u}}|$ and arrows indicate the flow direction

As a second example, we use the results of an isothermal numerical simulation ($\Delta T = 0, {\varvec{u}} \ne 0$) of the flow inside a rapidly rotating and precessing cylinder (Giesecke et al. 2024). The setup consists of a cylinder filled with a homogeneous fluid of a radius *R* and height $H=2R$ rotating around its axis of symmetry at an angular frequency $\Omega$ and precessing along an axis perpendicular to its rotation axis at $\Omega _p$. We run rescaled, dimensionless numerical simulations at Ekman Number $E=\frac{\nu }{\Omega r^2}=10^{-4}$ and Poincaré Number $Po=\frac{\Omega _p}{\Omega }=0.1$ using the kinematic viscosity $\nu =1.5\times 10^{-5}\, \frac{\text{ m}^2}{\text{ s }}$ of air and a radius $R=0.5$ m. The rotation and precession frequencies are, $\Omega = 0.1$ Hz and $\Omega _p = 0.01$ Hz, respectively. The resulting velocity field in a vertical cross section of the cylinder is shown in Fig. 3, exhibiting structures on a broad range of spatial scales with features of interest near the side walls, a notoriously challenging situation for ATOM.

**Fig. 4:**
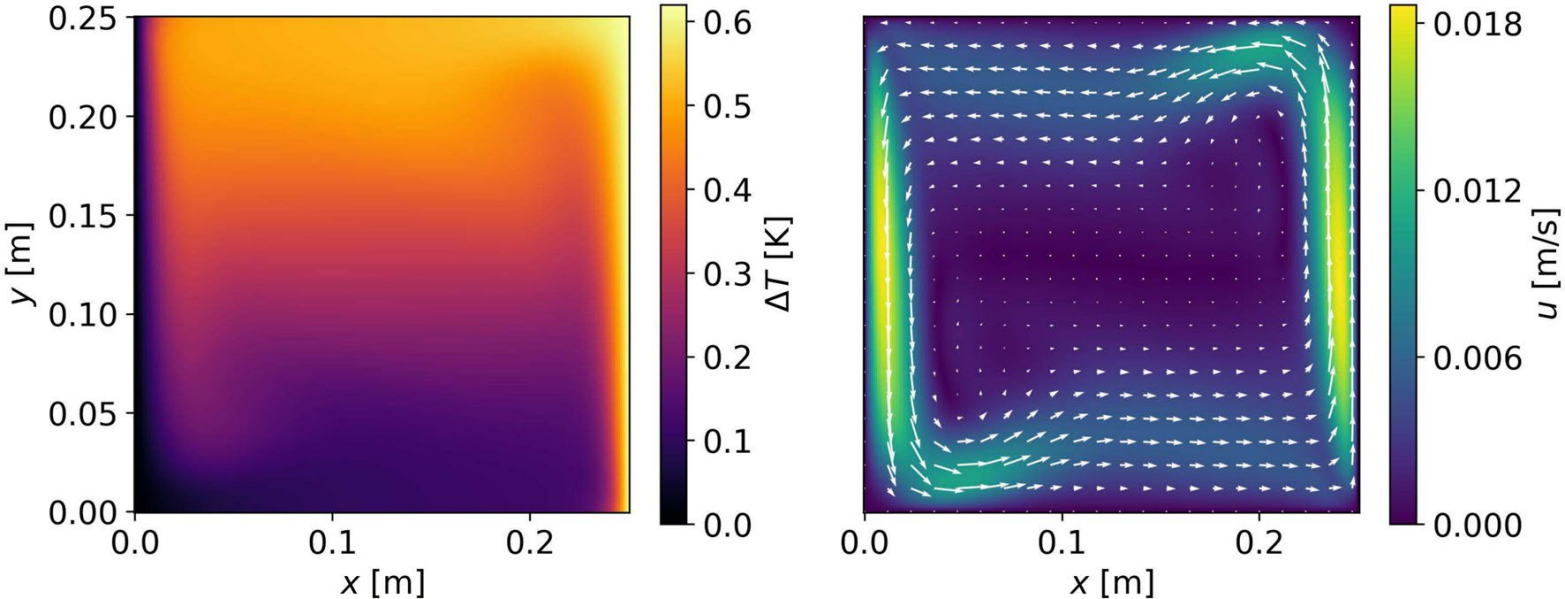
True temperature and flow field for the case of a convective Boussinesq-fluid after de Vahl Davis and Jones (1983). The displayed colors and arrows are analogous in meaning to the ones in the previous two figures

Finally, to test a joint temperature and flow inversion ($\Delta T \ne 0, {\varvec{u}} \ne 0$), we choose data of a convective simulation performed with the COMSOL Multiphysics software, in the setting posed by de Vahl Davis and Jones (1983). The temperature and flow field can be seen in Fig. 4.

The fields are solutions to the Navier–Stokes and energy equation in the Boussinesq approximation, where the dynamics are governed by buoyancy forces. The temperature on the right wall is set to $T = 300.62\,$K, while on the left wall, it is kept at $T_0$, yielding a Rayleigh Number of $Ra \approx 10^6$.

To discretize the model domain, we use a uniform Cartesian grid with $N \times N$ points separated by a grid step $\Delta h$. As a stability condition, it is required that $\Delta h \le \frac{1}{20}\,\lambda$, i.e., 20 grid steps per wavelength should be used to avoid any unwanted effects of grid dispersion (Alford et al. 1974). For a side length *D* of the domain, we choose the wavelength of the sound to be $\lambda = D/50$ and employ $N = 1536$ grid points per axis. This results in a grid step of $\Delta h \approx \frac{1}{30}\,\lambda$. In a practical application, it would correspond to an emission frequency $f_0\sim 17$ kHz in the first two cases, and $f_0\sim 70$ kHz for the horizontal convection case.

### Synthetic observed waveforms

To model the evolution of the sound pressure, we apply $p({\varvec{x}},0) = p({\varvec{x}},\Delta t) = 0$ at the first two time steps, to satisfy the initial conditions (27). From there on, the pressure field for each time step is computed iteratively by solving Eqs. (17) and (18) using explicit time stepping.

**Fig. 5:**
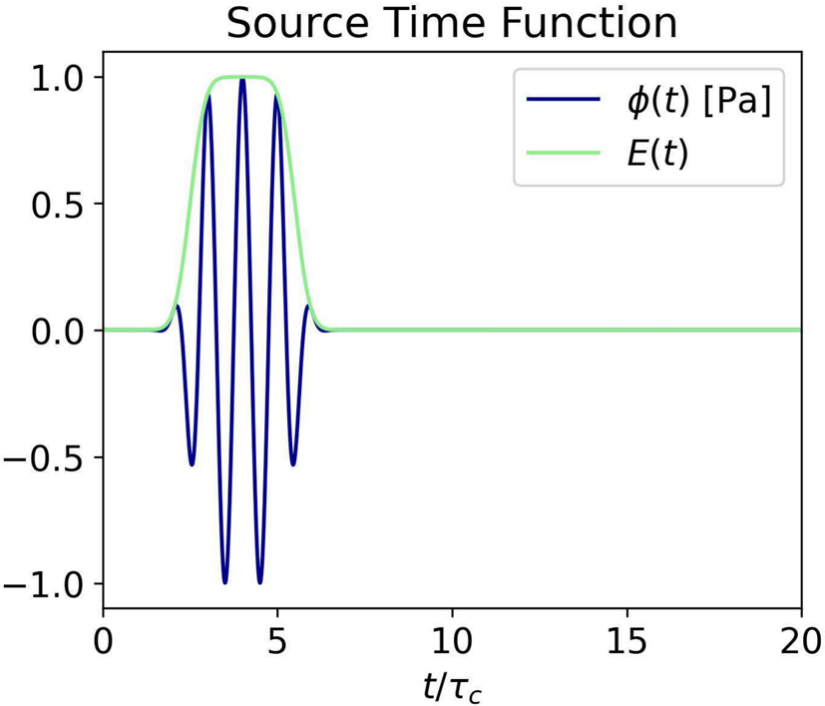
Source time function $\phi (t)$ and its envelope *E*(*t*)

To excite and record sound, we model transducers as single points on the boundary of the domain. We model the emitted signal as a cosine wavelet of central frequency $f_0 = 1/\tau _c$ modulated by an envelope *E*(*t*) which is constructed from two mirrored complementary error functions (erfc) centered around the time $t_0 = 4\,\tau _c$ (see Fig. 5). Thereafter, we refer to this as the source time function $\phi (t)$.

**Fig. 6:**
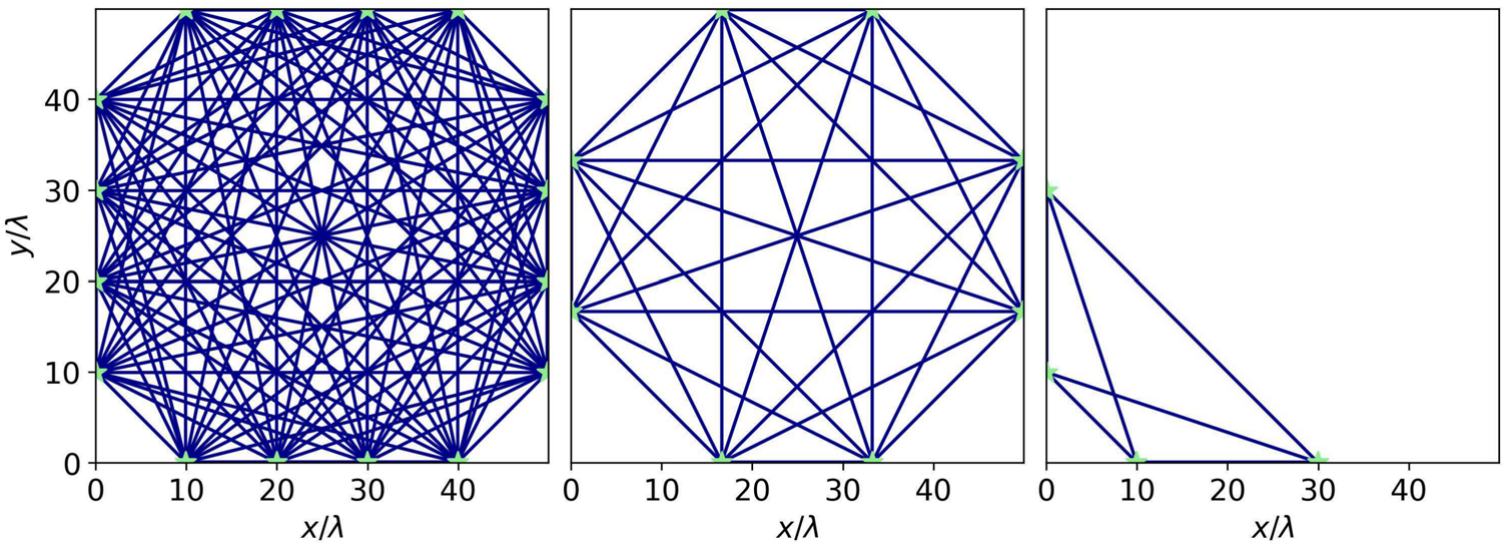
The three transducer arrays considered in this study. The sensors are marked with green stars and the direct rays between them are represented by blue lines

We employ three configurations of transducer positions in this study, shown in Fig. 6.

We refer to these, from left to right, as the $4\times 4$ -, $4\times 2$ - and $2 \times 2$ -Array.

One of the benefits of FWI is the feasibility of simultaneous emission at the same frequency for all transducer, hence offering the possibility to use simpler electronics with a single excitation circuit and a multiplexer. This will result in a small phase shift between the sources that we take into account by introducing a short delay of $0.5\,\tau _c$ between the transducers. This also prevents exact superposition of reflected waves when using perfectly symmetric arrangement of transducers.

The observed signals $p^\text {obs}(t)$ are generated by propagating the pressure signal from each transducer through the fluid domain to each receiver location, here we use transducers but the same will apply if emitters and receivers were at different locations.

**Fig. 7:**
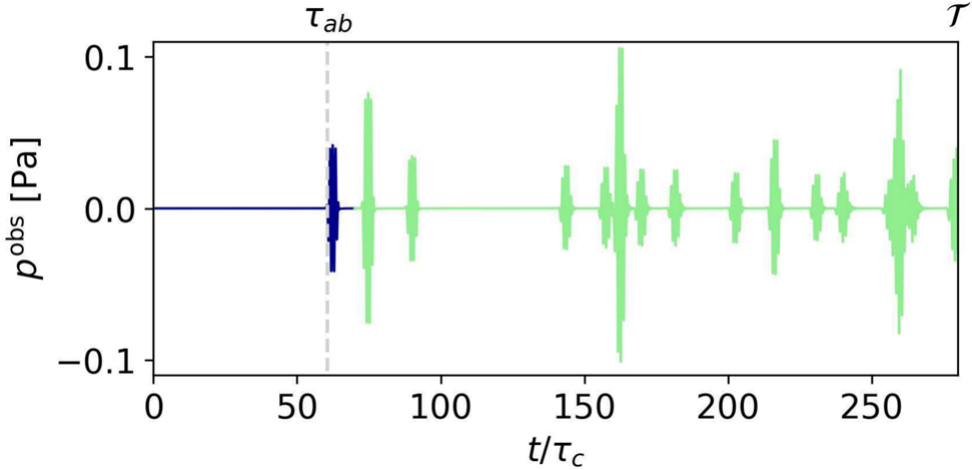
A synthetic waveform generated by the forward solver. It corresponds to the signal received by the rightmost transducer at the top boundary in the $4 \times 4$-sensor array, where the signal has been emitted by the leftmost transducer on the bottom boundary. $\tau _{ab}$ marks the time at which the first arrival (blue) is recorded. $\mathcal {T} = 280\,\tau _c$ is the signal duration we will consider, as it includes several reflected phases (green)

Since we can choose an arbitrary duration $\mathcal {T}$ for our measurements, there is no restriction on how many reflected phases we may consider in our receiver signals (see Fig. 7). For our simulations, we choose a time step of $\Delta t = 2\times 10^{-3}\,\tau _c$, to satisfy the CFL-criterion (Courant et al. 1928)

31 $$\begin{aligned} \text {max}\left[ \frac{c\,\Delta t}{\sqrt{2/\Delta h^2}}\right] < 1. \end{aligned}$$

In the case of the buoyancy-driven flow, we run our simulation with twice the temporal resolution to resolve the smaller time shifts introduced by the weaker flow.

### Prior model and adjoint simulations

For all reconstructions, our first guess is a homogeneous prior model with no flow and no temperature anomaly, i.e.,

32 $$\begin{aligned} {\varvec{m}}_0 = (c_0, 0, 0). \end{aligned}$$

This represents a scenario in which we have no á priori information about the fields we want to reconstruct.

Given that the operator $\mathcal {L}$ is self-adjoint, the adjoint simulations can be run using the forward solver. Here, time stepping has to be reversed and the simulations start with the terminal conditions $p^\dag ({\varvec{x}}, \mathcal {T}) = p^\dag ({\varvec{x}}, \mathcal {T}-\Delta t) = 0$, as customary with the adjoint method in the absence of viscous attenuation (Fichtner 2010).

### Gradient-descent

We come back to the step in Flowchart 1 of finding the optimal model update. Using a finite difference approximation of the model domain, a natural choice for optimization is a gradient descent. For this, the sensitivity kernel $K_{m_i}({\varvec{x}})$, sampled at the points of the grid, can be recast as a vector $\textbf{K}_{m_i}$ of $N^2$ elements. The *j*-th element is

33 $$\begin{aligned} \text {K}^j_{m_i} = K_{m_i}(x_j) = \frac{\partial \chi }{\partial \left[ m_i(x_j)\right] }, \end{aligned}$$

where $m_i(x_j)$ denotes one of the parameters *c*, $v_x$ or $v_y$ at the grid point $x_j$ (Fichtner (2010), Ch. 8.2.2). Treating the models as vectors $\textbf{m}_i$ with the elements $m_i(x_j)$, the optimization problem (25) can thus be reformulated as

34 $$\begin{aligned} \Delta \chi = \sum _i \textbf{K}_{m_i} \cdot \Delta \textbf{m}_i \end{aligned}$$

That is, we search the optimal model update $\Delta \textbf{m}_i$ for decreasing the objective function $\chi$ given the *gradient vector* $\textbf{K}_{m_i} = \nabla _{m_i}\chi$.

To solve this problem, we utilize a limited-memory BFGS method (see Nocedal and Wright (1999) for further details). This quasi-Newton method computes the step length and step direction of the model updates $\Delta \textbf{m}_{i,k}$ in each iteration *k* automatically from the information on gradients of previous iterations.

A measure to avoid an early departure into a local minimum of the misfit is smoothing the gradients for each iteration *k* with a two-dimensional Gaussian kernel of a defined standard deviation $\sigma _k$—we do this by projecting the gradient vector back onto the two-dimensional grid. We start with a wide kernel of $\sigma _0 = 5\,\lambda$ and increase the degree of detail by reducing the width throughout the inversion down to, typically, half a wavelength.

### Straight-ray inversion benchmark algorithm

To outline the benefits and limitations of the FWI with respect to conventional straight-ray algorithms, we also perform inversions for the same synthetic temperature and flow fields using the freely available straight-ray code of Wiens and Behrens (2009). In the ray approximation, the input data for the inversion are the travel times between each emitter and receiver. It is not necessary to propagate the waveform in the domain, but simply to calculate the time of arrival by integrating the effect of advection and sound speed variation along the ray path, as described by Eq. (3).

There exist many algorithms to invert travel times for velocity and temperature fields. Wiens and Behrens (2009) adopt an approach using Gaussian radial basis functions (RBFs). The positions of the RBF-centers are chosen randomly, while the number $N_\text {RBF}$ of Gaussian RBFs and their width $\sigma _\text {RBF}$ can be freely chosen.

Since the effect of these two parameters on the quality of the inversions is not discussed in Wiens and Behrens (2009), we test all combinations of $N_\text {RBF} \in \{25, 50, 100, 200, 400\}$ and $\sigma _\text {RBF} \in \{1, 2, 3, 5, 8, 9, 12, 15, 18, 25\}\,\lambda$.

For each combination $(N_\text {RBF}, \sigma _\text {RBF})$, we carry 30 straight-ray inversions (SRIs), each corresponding to a different random realization of the RBF-centers’ positions. We assess the quality of each inversion using the root relative square error (RRSE). The RRSE of the temperature reconstructions is computed as

35 $$\begin{aligned} E(\Delta T) = \sqrt{\frac{\sum _j \left( \Delta T(x_j) - \Delta T_\text {true}(x_j)\right) ^2}{\sum _j \left( \Delta T_\text {true}(x_j) - \overline{\Delta T}_\text {true}\right) ^2}}, \end{aligned}$$

for a true and estimated model $\Delta T_\text {true}$ and $\Delta T$, respectively, where $\overline{\Delta T}_\text {true}$ is the average of the former across all grid points $x_j$. Analogously, the RRSE of a flow reconstruction is

36 $$\begin{aligned} E({\varvec{u}}) = \sqrt{\frac{\sum _j \left| {\varvec{u}}(x_j) - {\varvec{u}}_\text {true}(x_j)\right| ^2}{\sum _j \left| {\varvec{u}}_\text {true}(x_j) - \overline{{\varvec{u}}}_\text {true}\right| ^2}}\,. \end{aligned}$$

We form the average of the RRSE over the 30 random realizations for each combination $(N_\text {RBF}, \sigma _\text {RBF})$. The optimal combination with the smallest mean error is used for a reconstruction to compare to our FWI.

To be fully consistent, we average the results of the FWI linearly onto the same grid as for the SRI, i.e., on $128 \times 128$ regularly spaced points.

In the next section, we will demonstrate the benefits of the FWI over a simpler ray-based ATOM. It should be noted that the increased information recovered from FWI comes at a significantly higher computational cost. We typically have to perform up to 25 iterations, each involving a propagation of the forward and adjoint wavefield.^1^ A complete reconstruction with our non-optimized educational code takes up to 8 h, including the generation of synthetic observed data. In contrast, the RBF algorithm of Wiens and Behrens (2009) takes only a few seconds to perform an inversion including the generation of the synthetic times of arrival.

## Results

In Fig. 7, we show a typical waveform $p^\text {obs}(t)$ sampled at a receiver ${\varvec{x}}_b$ resulting from the emission by a single emitter ${\varvec{x}}_a$ as obtained from our forward solver.

The information extracted by a ray-based code incorporating only first arrivals would merely be the time of first arrival, $\tau _{ab}$. In contrast, we can simulate and invert the entire waveform up to $\mathcal {T}$ including not only the later arrivals but also the shape of the signals. That is, we do not have to know which arrival corresponds to which ray path and we do not have to avoid overlapping arrivals to ensure the practicability of a cross-correlation. This is a crucial benefit when using simultaneously emitting sources and dealing with much less orderly signals as the one displayed in Fig. 7.

### Temperature reconstruction

**Fig. 8:**
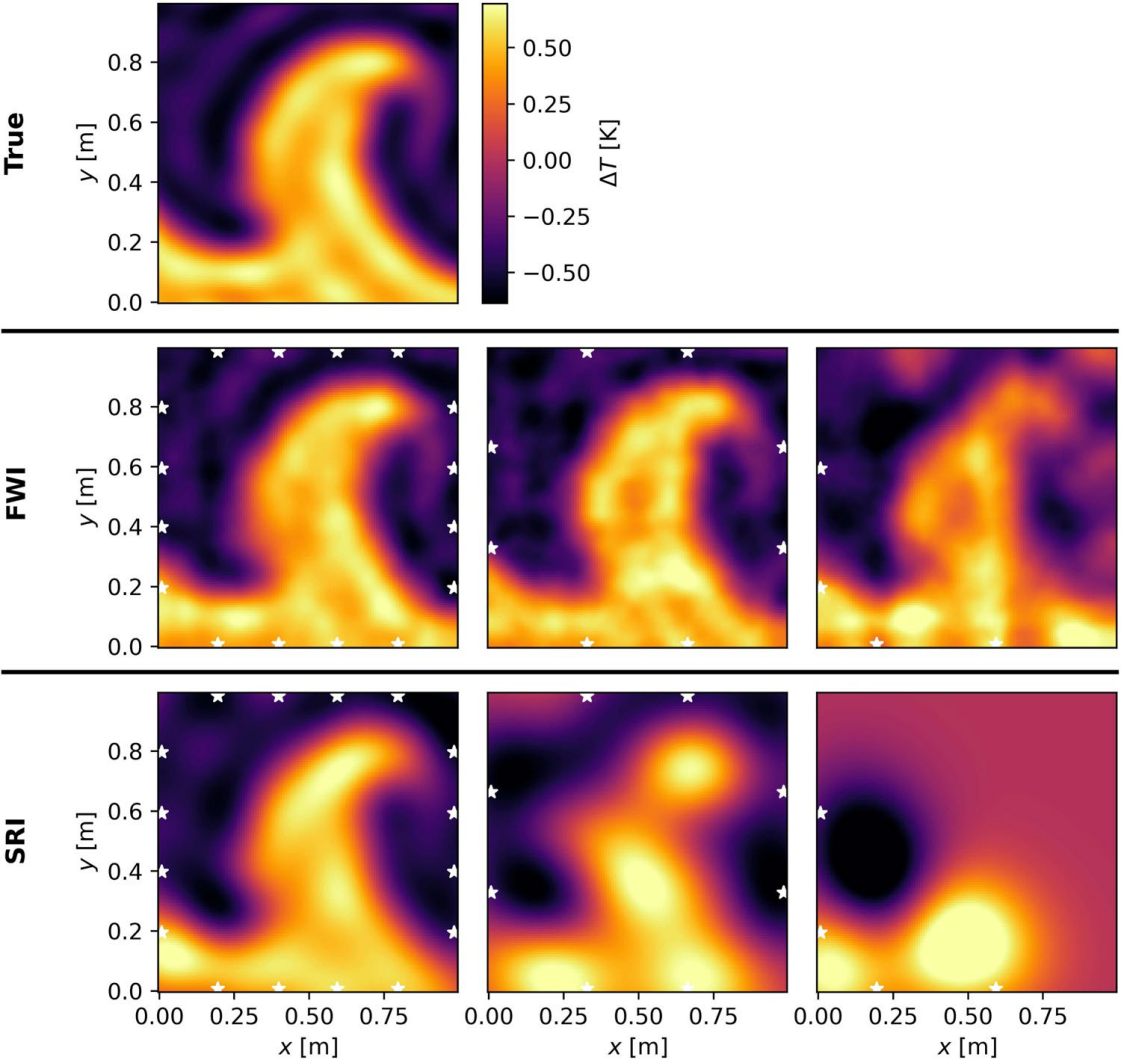
Upper: True temperature anomaly of the model provided by Wiens and Behrens (2009). Middle: Reconstructions by our full-waveform algorithm for the three different sensor arrays—$4\times 4$, $4\times 2$ and $2\times 2$, from left to right. Lower: Reconstructions by the straight-ray algorithm, analogous to the row above. The white stars indicate the transducer positions

We first use the dataset of Wiens and Behrens (2009) to assess the feasibility of reconstructing the temperature anomaly in the absence of any flow. The true temperature anomaly and the reconstructions by the FWI and SRI are shown in Fig. 8.

It can be seen that for the largest number of sensors, both methods can robustly capture the shape of the plume. However, it is clear that, as the number of sensors is reduced, we can recover less and less useful information using the SRI. This can be easily understood from the decimation of the ray path coverage as illustrated in Fig. 6. To assess the quality of the inversions, we calculate the RRSE between the true and each reconstructed temperature field. The errors are listed in Table 1.

**Table 1:** RRSEs of the full-waveform and straight-ray inversions of the temperature anomaly using all three considered transducer arrays

Array	$E(\Delta T_\text {FWI}) (\%)$	$E(\Delta T_\text {SRI}) (\%)$
$4 \times 4$	10	18
$4 \times 2$	17	42
$2 \times 2$	42	82

The errors show what has already been anticipated from observing Fig. 8. That is, the FWI is less prone to erroneous reconstructions when decreasing the number of sensors, harnessing the information carried by late arrivals of reflected waves sampling a much larger region of the fluid domain.

### Velocity reconstruction

**Fig. 9:**
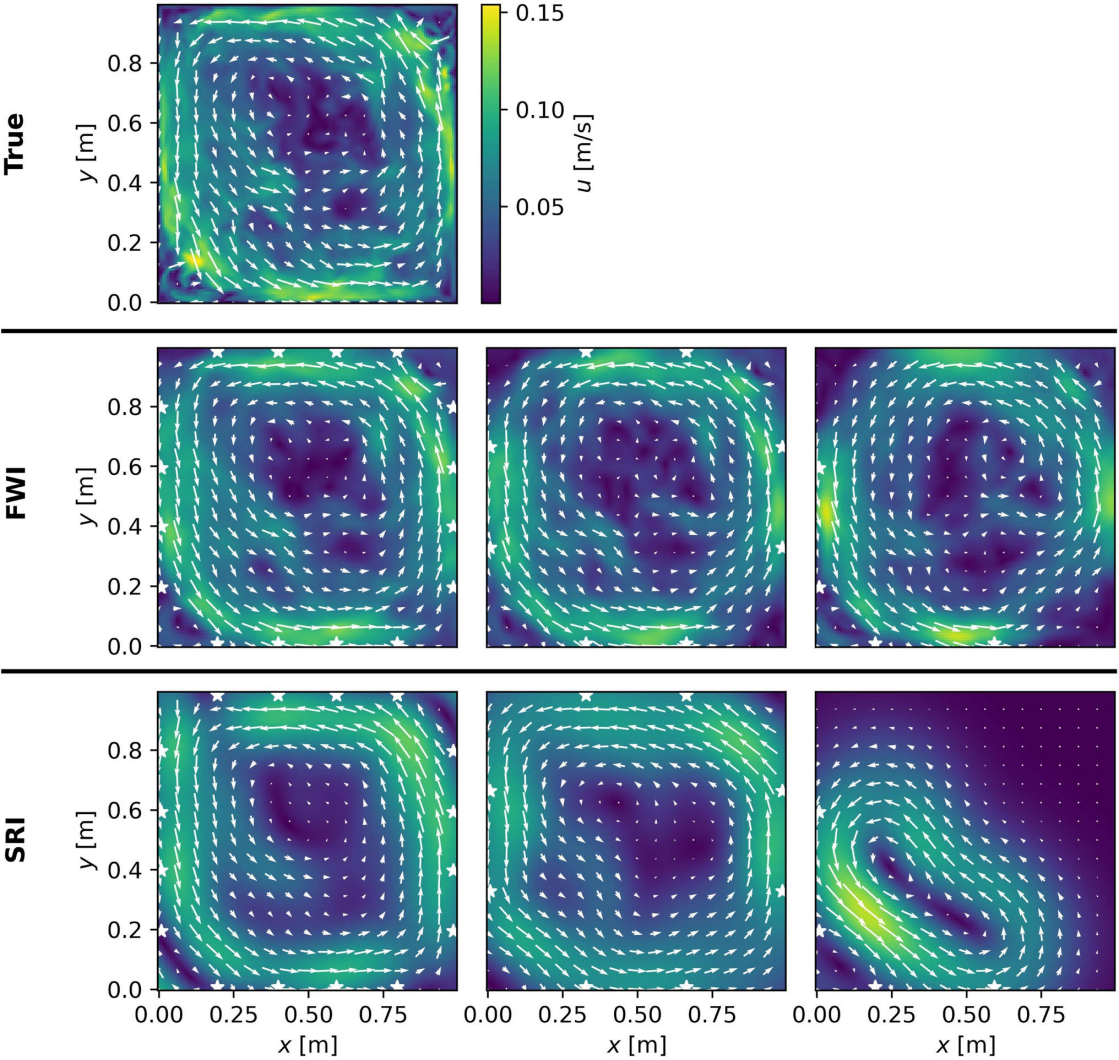
Upper: True velocity field of the precession driven, isothermal flow. Middle: Reconstructions by the FWI for the three different sensor arrays—$4\times 4$, $4\times 2$ and $2\times 2$, from left to right. Lower: Reconstructions by the straight-ray algorithm, analogous to the row above. White stars denote the transducers

The reconstructed isothermal velocity fields in the precessing cylinder are shown in Fig. 9, for both the FWI and the SRI.

We see that the FWI manages to recover the large-scale, counter-clockwise flow as well as some smaller-scale features along the walls and in the center of the domain. This is true for all three arrays considered. The SRI can recover the large-scale flow in the case of 16 and eight sensors, although with a systematic and significantly larger error than the FWI. In the case of four sensors, the information along the direct rays is not sufficient to recover any of the flow features.

We again compare the RRSEs between the two methods in Table 2.

**Table 2:** RRSEs of the full-waveform and straight-ray inversions of the isothermal flow for each considered transducer arrays

Array	$E({\varvec{u}}_\text {FWI}) (\%)$	$E({\varvec{u}}_\text {SRI}) (\%)$
$4 \times 4$	27	40
$4 \times 2$	36	47
$2 \times 2$	47	102

Although we note higher values of the RRSE for both methods as for the previous temperature reconstruction—which may be resulting from an added degree of freedom, given we are reconstructing vector quantities—the trend is similar. The FWI can make use of the additional information in the waveform data, to offset the increasing sparsity of the arrays. That is, as we have observed before, when using only the four sensors in the bottom left corner, the FWI performs as well as the SRI using eight sensors all around the domain.

### Joint reconstruction of temperature and flow

**Fig. 10:**
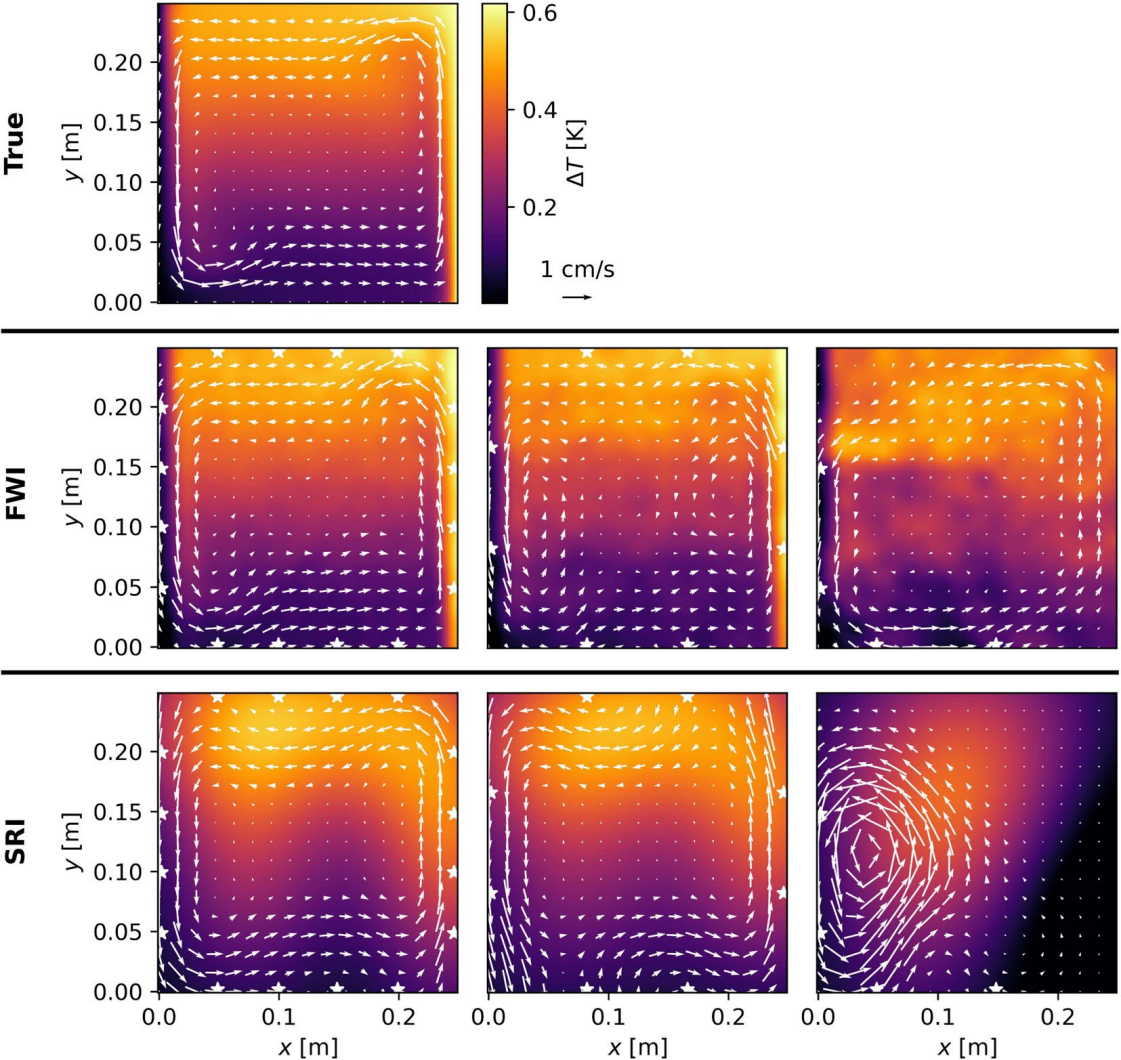
Upper: True temperature and velocity field associated to the buoyancy-driven flow, where the arrows display direction and magnitude of the flow. Middle and Lower: Reconstructions by the FWI and SRI, respectively, analogous to the reconstructions depicted in the previous two figures

The results of the joint temperature and flow reconstructions are shown in Fig. 10.

The great challenge of this reconstruction comes from the mixed effects of temperature and flow fields. A clear benefit of the SRI is the ability to separate temperature and flow effects using the sum and difference of reciprocal travel times—as outlined in Eqs. (4) and (5), respectively. Although this is not implemented in the algorithm, we use by Wiens and Behrens (2009).

For the FWI, there is no equivalent to these equations. Hence, the joint reconstruction provides a critical test for our method.

To address the problem of any cross-talk between the flow and sound speed anomalies in the FWI, we separately and alternately update the temperature and velocity field—starting with the temperature, as the phase shift introduced by it is larger than the one introduced by the velocity^2^. After a few iterations, updating only the temperature, we observe the misfit’s decrease becoming marginal, at which point we start updating the flow until we observe the same phenomenon and repeat the process until full convergence is achieved. Note that this strategy has no impact on how fast the data can be acquired but only on the inversion. Table 3 lists the RRSEs of the joint reconstructions displayed in Fig. 10.

**Table 3:** RRSEs of the full-waveform and straight-ray inversions of the convective flow for the three considered transducer arrays

Array	$E(\Delta T_\text {FWI}) (\%)$	$E(\Delta T_\text {SRI}) (\%)$	$E({\varvec{u}}_\text {FWI}) (\%)$	$E({\varvec{u}}_\text {SRI}) (\%)$
$4 \times 4$	8	39	39	48
$4 \times 2$	12	42	49	66
$2 \times 2$	40	139	66	126

Here, we observe a large discrepancy between the qualities of the recovered temperature models by the FWI and the SRI, even for the $4 \times 4$- and $4 \times 2$-Array. Looking at the reconstructions, we note that the FWI detects the sharp thermal boundary layers at the side walls, while the ray-based reconstruction is very smooth all over the domain, showing no evidence of a thermal boundary layer. The reconstructions of the temperature and velocity using the $2 \times 2$-Array for the SRI fail to capture the dynamics of the system, similarly to what has been reported in the two previous examples.

## Discussion

**Fig. 11:**
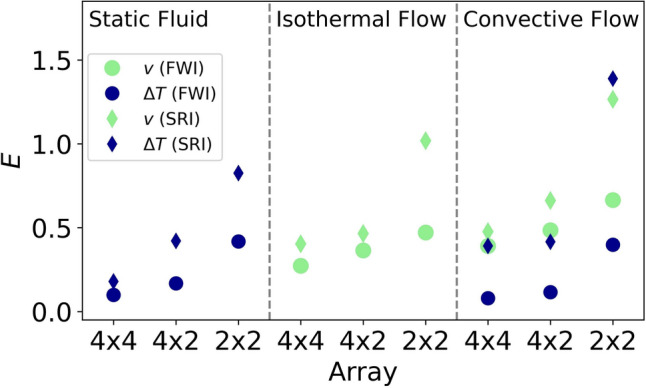
Overview on the RRSEs of all obtained reconstructions. The data points labeled with $\Delta T$ and *v* refer to temperature and flow reconstructions, respectively

Figure 11 summarizes the RRSEs of the models for all discussed configurations of the full-waveform and straight-ray reconstructions.

The primary insight from this graphic is the following. When the number and locations of the sensors allow for a good coverage of the domain by direct rays, both FWI and SRI perform similarly well. That is, a straight-ray algorithm—being computationally more efficient— might be the preferred choice for an inversion if a dense coverage of direct rays can be achieved. The only exception is the temperature anomaly of the horizontal convection, where the SRI fails at capturing the thermal boundary layers. However, in the code of Wiens and Behrens (2009) the regularization is controlled by the fixed width of the Gaussian RBFs, which sets the length scale of the temperature gradients throughout the entire domain. That is, the inversion can hardly produce both large-scale features and sharp gradients in a single reconstructed model. This might be fixed by choosing a different technique of regularization.

Meanwhile, when the ray coverage is slightly reduced, the quality of the reconstructions by the straight-ray algorithm starts to decay, resulting from a lack of information. The FWI, which can take into account multiple reflections, does generally not experience any significant increase in the model error.

Even for the $2 \times 2$-sensor array, where an inversion based on direct rays is practically impossible, the FWI can still adequately reconstruct the main features of the temperature and velocity fields throughout the entire domain.

Thus, the FWI presents itself as a valuable tool to overcome under-determinedness when the positioning of the transducers does not allow for an adequate coverage of direct rays inside an enclosed domain, a problem outlined as a key challenge in ATOM (Othmani et al. 2023).

A second advantage of the newly developed method is its capability to reduce the acquisition time by inverting simultaneously emitted signals without a stringent constraint on the source time function. A ’frequency division’ or ’colored frequency’ approach, as the one used by Wiens and Behrens (2009), is likely to fail when considering reflected signals in a laboratory size, enclosed domain—as discussed in Sect. 1.1.

On the other hand, if the enclosed flow would be probed by consecutively emitted signals, one might have to wait to activate each source until *all reflected waves* excited by the previously activated source have significantly decayed, to avoid interference with the newly emitted signal. This makes separate emission—on which most ray-based methods rely—even less desirable than in free domains and might render scans of rapidly changing, turbulent flows impractical in enclosed domains.

To our knowledge, the FWI is thus far the only proposed algorithm to handle both simultaneous emission and the incorporation of later arrivals to reconstruct flow and temperature fields in fluid media.

While, from the hardware standpoint several benefits of the FWI are already demonstrated, there is still potential for improving on the algorithm’s computational cost and accuracy.

Firstly, the $L_2$-misfit we utilize for this exploratory study is widely considered as nonoptimal, where Fichtner (2010) (Ch. 11.2), e.g., argues that it hyper-focuses on large amplitude waveform differences and omits information carried by phase shifts between low amplitude waves. Alternative misfit functions may for instance reduce cross-talk in the joint reconstruction of temperature and velocity and the need to alternately update the models might be eliminated. Since flow reconstructions are a largely unexplored application of FWI, research on a fitting misfit for ATOM may be vital for further improving its practicability.

Secondly, our finite difference code is only suited for Cartesian boxes. Applying the Neumann condition (18) is a challenge when modeling boundaries other than that of a rectangular domain using the finite difference approximation. However, recent progress in full-waveform modeling has already brought up promising alternatives, such as spectral element modeling (e.g., Komatitsch and Tromp (2002a, 2002b); Peter et al. (2011); Afanasiev et al. (2019)), which provides accurate solutions for two- and even three-dimensional domains efficient enough to be run on a desktop computer or a single core of a laptop (Afanasiev et al. 2019).

Spectral element methods allow for the usage of arbitrarily shaped model boundaries (Afanasiev et al. 2018) and might facilitate inversions for flows by utilizing the reflected signals back-scattered by, e.g., objects in wind tunnels or lake-bottom tomography. Imaging flows around obstacles requires advanced techniques and is a current field of research (Wieneke and Rockstroh 2024; Hendriksen et al. 2024). Thus, it will be worthwhile to test how well acoustic FWI, by simply adjusting the shape of the boundary, can reconstruct such flows.

Finally, further development is needed for this approach to be applied in open domains, i.e., in the absence of solid boundaries. In seismology, there already exist several solutions to this problem (see, e.g., Fichtner (2010) Ch. 6). Thus, further research should focus on generalizing these results to the case of a nonzero flow field and the absence of the non-penetration condition at the boundary.

Throughout this study, we have consistently tested the FWI against the SRI in the ideal case of noise-free data. Moving toward real measurements, the logical next step is assessing the effect of different noise levels on the reconstructions for both methods. Another general challenge is that the transfer functions of the probes and the electronics connected to them may be unknown. The implemented source time function in the FWI code must precisely match the output signal of the hardware in order for the estimated waveforms to be meaningful. A calibration in a static, isothermal fluid might be the best solution in this case. Additionally, transducers may have to be modeled as finite sources instead of single points.

Given its heavy computational cost, the FWI can be used to reconstruct individual snapshots of flows rather than tracking their temporal evolution. Furthermore, since the computational effort increases with the number of wavelengths along which the sound has to be propagated, applying FWI for flow measurements in large domains, such as in ocean or atmospheric tomography, naturally represents a challenge. With respect to these restrictions, it is worth closely following the work of Schade et al. (2024) who research on speeding up wave simulations using quantum computers.

## Conclusion

This study has outlined the potential benefits provided by FWI for reconstructing laboratory-scale flows using acoustic tomography. The ability to invert for simultaneously emitted signals from all sources can significantly decrease the acquisition time in enclosed domains. Additionally, waves that are back-scattered from the domain boundaries are naturally included in the inversion, increasing the amount of available data and, eventually, the reconstruction quality when restricted to a sparse transducer array.

This might be especially useful when imaging flows around objects or topography on which no sensor can be mounted.

We highlight that our exploratory code is not yet optimized and leaves room for improvement. This includes the replacement of the $L_2$-misfit with an alternative more suited for joint temperature and flow inversions as well as a new approximation of the governing equations. That is, our finite difference approach shows drawbacks in both efficiency and implementing complex domain geometries.

As a final prospect, we would like to highlight that almost no added modeling complexity may be needed to invert for the sound speed and flow in a multiphase fluid. As the primary model parameters in the inversion are *c* and ${\varvec{u}}$, this algorithm might as well be utilized to invert for the sound speed distribution in, e.g., a scenario where droplets of oil or metal are suspended in water. The FWI is known to detect such sharp material boundaries in seismology (e.g., Virieux et al. (2017)).

## Supplementary Information

Below is the link to the electronic supplementary material.

Supplementary material containing a detailed derivation of the forward wave equation and the adjoint wave operator

A folder including the data and Python notebook to reproduce Figures 8 to 11. Additionally, we provide the regularization parameters applied for the straight-ray inversions ($N_\text {RBS}$ and $\sigma _\text {RBF}$) and full-waveform inversions (width $\sigma _k$ of Gaussian smoothing kernels used in each iteration).

## Data Availability

The data essential to reproducing the figures in the Results section are submitted together/published alongside this article. The routines for running the full-waveform simulations are openly accessible: https://github.com/lennartKira/Full-Waveform-Acoustic-Tomography-for-moving-Fluids For receiving any uncompressed, large files created by the full-waveform algorithm, please contact the corresponding author. The Python library *Pestoseis* can be found using the following link: https://github.com/inverseproblem/PestoSeis. The original code of Travis Wiens can be found on his repository: https://www.mathworks.com/matlabcentral/fileexchange/22174-rbf-acoustic-tomography Instructions and data to reproduce the buoyancy-driven convection model through COMSOL can be accessed here: https://www.comsol.com/model/buoyancy-flow-of-free-fluids-665

## Footnotes

1 It is hard to define quantitative criteria for the convergence of FWI (e.g., Marty et al. (2021)), here we stop when there is no significant decrease of the misfit or the appearance of unnatural small scale features close to the sensors.

2 This can be estimated á priori by approximating the integral (3) using the typical length, temperature and flow scales of the problem.
