# Supplementary material for: Full-waveform acoustic tomography for fluid temperature and flow
Source: Exp Fluids. 2025 Jul 8;66(8):145. doi: 10.1007/s00348-025-04068-z (PMC12238201; doi:10.1007/s00348-025-04068-z)
Supplement: Supplementary file 1 — Supplementary material containing a detailed derivation of the forward wave equation and the adjoint wave operator [file 348_2025_4068_MOESM1_ESM.pdf]

# Full-Waveform Acoustic Tomography for Fluid Temperature and Flow: Supplementary Material

Lennart Kira[1*] and Jerome Noir[1]

[1]Department of Earth and Planetary Sciences, ETH Zürich, Sonneggstrasse 5, Zurich, 8090, Switzerland.

*Corresponding author(s). E-mail(s): lennart.kira@eaps.ethz.ch; Contributing authors: jerome.noir@eaps.ethz.ch;

## Derivation of the Forward Wave Equation

To derive the forward operator, i.e., the wave equation, we first start with the perturbed Navier-Stokes Equations (11) and (12) in the main text.

$$\rho' \left( \frac{\partial}{\partial t} + \boldsymbol{u}' \cdot \nabla \right) \boldsymbol{u}' = -\nabla p' + \rho' \nu \nabla^2 \boldsymbol{u}'$$

$$\left( \frac{\partial}{\partial t} + \boldsymbol{u}' \cdot \nabla \right) \rho' = -\rho' \, \nabla \cdot \boldsymbol{u}' \,,$$

where $\boldsymbol{u}' = \boldsymbol{u} + \tilde{\boldsymbol{u}}$, $p' = p + \tilde{p}$ and $\rho' = \rho + \tilde{\rho}$.

If we subtract the equations of the background flow - Equations (6) and (7) in the main text - from these, we obtain

$$\rho' \left[ \frac{\partial}{\partial t} \tilde{\boldsymbol{u}} + \tilde{\boldsymbol{u}} \cdot \nabla \tilde{\boldsymbol{u}} + \boldsymbol{u} \cdot \nabla \tilde{\boldsymbol{u}} + \tilde{\boldsymbol{u}} \cdot \nabla \boldsymbol{u} \right] + \tilde{\rho} \frac{D}{Dt} \boldsymbol{u} = -\nabla \tilde{p} + \rho' \nu \nabla^2 \tilde{\boldsymbol{u}} + \tilde{\rho} \nu \nabla^2 \boldsymbol{u} \tag{1}$$

$$\frac{D}{Dt} \tilde{\rho} + \tilde{\boldsymbol{u}} \cdot \nabla (\rho + \tilde{\rho}) = -\rho' \nabla \cdot \tilde{\boldsymbol{u}}. \tag{2}$$

Now, we write the velocity perturbation in terms of the wave displacement: $\tilde{\boldsymbol{u}} = \left( \frac{\partial}{\partial t} + \boldsymbol{u} \cdot \nabla \right) \boldsymbol{\xi}$. Substituting this into the momentum equation yields the lengthy equation

$$\begin{aligned} (\rho + \tilde{\rho}) & \left[ \partial_t^2 \boldsymbol{\xi} + (\partial_t \boldsymbol{u} \cdot \nabla) \boldsymbol{\xi} + 2 \boldsymbol{u} \cdot \nabla \partial_t \boldsymbol{\xi} + (\partial_t \boldsymbol{\xi}) \cdot \nabla \partial_t \boldsymbol{\xi} + (\boldsymbol{u} \cdot \nabla \boldsymbol{\xi}) \cdot \nabla \partial_t \boldsymbol{\xi} + \partial_t \boldsymbol{\xi} \cdot \nabla (\boldsymbol{u} \cdot \nabla \boldsymbol{\xi}) \right. \\ & \left. + (\boldsymbol{u} \cdot \nabla \boldsymbol{\xi}) \cdot \nabla (\boldsymbol{u} \cdot \nabla \boldsymbol{\xi}) + \boldsymbol{u} \cdot \nabla (\boldsymbol{u} \cdot \nabla \boldsymbol{\xi}) + (\partial_t \boldsymbol{\xi} \cdot \nabla) \boldsymbol{u} + (\boldsymbol{u} \cdot \nabla \boldsymbol{\xi}) \cdot \nabla \boldsymbol{u} \right] + \tilde{\rho} (\partial_t \boldsymbol{u} + \boldsymbol{u} \cdot \nabla \boldsymbol{u}) \\ & = -\nabla \tilde{p} + (\rho + \tilde{\rho}) \left[ \nu \nabla^2 (\partial_t \boldsymbol{\xi} + \boldsymbol{u} \cdot \nabla \boldsymbol{\xi}) \right] + \tilde{\rho} \nu \nabla^2 \boldsymbol{u}. \end{aligned} \tag{3}$$

Furthermore, the continuity equation becomes

$$\partial_t \tilde{\rho} + \boldsymbol{u} \cdot \nabla + (\partial_t \boldsymbol{\xi}) \cdot \nabla (\rho + \tilde{\rho}) + (\boldsymbol{u} \cdot \nabla \boldsymbol{\xi}) \cdot \nabla (\rho + \tilde{\rho}) = -(\rho + \tilde{\rho}) \left[ \nabla \cdot \partial_t \boldsymbol{\xi} + \nabla \cdot (\boldsymbol{u} \cdot \nabla \boldsymbol{\xi}) \right]. \tag{4}$$

To estimate the scale of each term, we introduce dimensionless variables

$$\begin{aligned} \boldsymbol{x}^* = \frac{1}{\lambda} \boldsymbol{x}, \quad & t^* = \frac{c_0}{\lambda} t, \\ \boldsymbol{\xi}^* = \frac{1}{\varepsilon} \boldsymbol{\xi}, \quad p^* = \frac{1}{\Pi} \tilde{p}, \quad & \rho^* = \frac{1}{\zeta} \tilde{\rho}, \\ \boldsymbol{u}^* = \frac{1}{u_0} \boldsymbol{u} \quad \text{and} \quad & \Delta T^* = \frac{1}{\Delta T_0} \Delta T, \end{aligned} \tag{5}$$

which we assume to be of unit size in the dynamical regime of the sound wave. Here, $\lambda$ is the typical wavelength of the acoustic waves, $c_0$ is the sound speed of the medium at background temperature, $\varepsilon$ is the typical amplitude of particle motion. $\Pi$ and $\zeta$ are the typical pressure and density perturbations, respectively, introduced by the wave motion. $u_0$ and $\Delta T_0$ are the typical background flow speed and temperature anomaly.
We assume that the background fields - the velocity and the temperature anomaly - possess a larger length scale $L$ and a longer time scale $\frac{L}{v_0}$ than the sound wave. That is, they are functions of $\frac{\lambda}{L}\boldsymbol{x}^*$ and $\frac{\lambda v_0}{L c_0}t^*$. Inserting these variables into Equations (3) and (4) yields

$$\left[1+\alpha\Delta T_0\,\Delta T^*+\frac{\zeta}{\rho_0}\rho^*\right]\left(\partial^2_{t^*}\boldsymbol{\xi}^*+2Ma\,\boldsymbol{u}^*\cdot\nabla^*\partial_{t^*}\boldsymbol{\xi}^*+\mathcal{O}\left(Ma\frac{\lambda}{L},\,\frac{\varepsilon}{\lambda},\,Ma^2,\,\frac{\tau_c}{\tau_\nu},\ldots\right)\right)=-\frac{\lambda\,\Pi}{\varepsilon\rho_0 c_0^2}\nabla^*p^* \tag{6}$$

and

$$\begin{aligned}&\frac{\lambda\zeta}{\varepsilon\rho_0}\partial_{t^*}\rho^*+\frac{\zeta}{\rho_0}\partial_{t^*}\boldsymbol{\xi}^*\cdot\nabla^*\rho^*+Ma\frac{\zeta}{\rho_0}\boldsymbol{u}^*\cdot\nabla^*\boldsymbol{\xi}^*\cdot\nabla^*\rho^*+Ma\frac{\lambda\zeta}{\varepsilon\rho_0}\\&=-\left[1+\alpha\Delta T_0\Delta T^*+\frac{\zeta}{\rho_0}\rho^*\right](\nabla^*\cdot\partial_{t^*}\boldsymbol{\xi}^*+Ma\nabla^*\cdot(\boldsymbol{u}^*\cdot\nabla^*\boldsymbol{\xi}^*),)\end{aligned} \tag{7}$$

where we have defined the Mach Number $Ma = u_0/c_0$, the acoustic timescale $\tau_c = \lambda/c_0$ and the viscous time scale $\tau_v = \lambda^2/\nu$.
In the momentum equation, the term $\partial^2_{t^*}\boldsymbol{\xi}^*$ is definitely of unit size and only the term on the right hand side can possibly balance it. This means, that with

$$\frac{\lambda\,\Pi}{\varepsilon\rho_0 c_0^2}=1\Rightarrow\varepsilon=\frac{\lambda\,\Pi}{\rho_0 c_0^2} \tag{8}$$

we can estimate the scale of the wave amplitude $\varepsilon$ if we know the sound pressure $\Pi$.
Also, the continuity equation only contains one term $\nabla^*\cdot\partial_{t^*}\boldsymbol{\xi}^*$ of definite unit size. If we assume $\zeta << \rho_0$, then the only candidate to balance this term is $\frac{\lambda\zeta}{\varepsilon\rho_0}\partial_{t^*}\rho^*$. That is, we can relate the scale $\zeta$ of the density perturbation to the pressure level by

$$\frac{\lambda\zeta}{\varepsilon\rho_0}=1\Rightarrow\zeta=\frac{\varepsilon\rho_0}{\lambda}\Rightarrow\zeta=\frac{\Pi}{c_0^2}. \tag{9}$$

Now, let us estimate some typical scales as would be present in a real experiment. Assume probing a domain filled with air ($\nu = 1.5\times10^{-5}\,\frac{\mathrm{m}^2}{\mathrm{s}}$, $c_0 = 346\,\frac{\mathrm{m}}{\mathrm{s}}$, $\rho_0 = 1\,\frac{\mathrm{kg}}{\mathrm{m}^3}$ and $\alpha = 3.3\times10^{-3}\,\frac{1}{\mathrm{K}}$) and a flow and temperature field with a typical length scale of $L = 1\,\mathrm{m}$. With a typical flow velocity of $u_0 = 10\,\frac{\mathrm{cm}}{\mathrm{s}}$, a sound pressure level of $\Pi = 1\,\mathrm{Pa}$ (corresponding to 94 dB) and probing the flow with a wavelength of $\lambda = 2\,\mathrm{cm}$ (corresponding to a central frequency of $f_0 = \frac{c_0}{\lambda} \approx 17\,\mathrm{kHz}$). We obtain $\varepsilon \approx 10^{-7}\,\mathrm{m}$ and $\zeta = 10^{-5}\,\frac{\mathrm{kg}}{\mathrm{m}^3}$. If assuming a temperature anomaly of $\Delta T_0 = 1\,\mathrm{K}$ we may neglect all terms of $\mathcal{O}\left(Ma\approx10^{-4}\right)$ or larger in the momentum equation to obtain

$$\left[\frac{\partial^2}{\partial {t^*}^2}\boldsymbol{\xi}^*+2Ma\,\boldsymbol{u}^*\cdot\nabla^*\frac{\partial}{\partial t^*}\boldsymbol{\xi}^*\right]=-\frac{1}{1+\alpha\Delta T_0\,\Delta T^*}\nabla^*p^*. \tag{10}$$

Note that $Ma$ is much smaller than unity and thus, the advection term is retained as a second order term. We may now introduce the material equation

$$\tilde{p}=-\rho c^2(\Delta T)\nabla\cdot\boldsymbol{\xi} \tag{11}$$

which can be formulated in dimensionless form using (8),

$$p^*=-(1+\alpha\Delta T_0\,\Delta T^*)\,{c^*}^2(\Delta T^*)\,\nabla^*\cdot\boldsymbol{\xi}^*, \tag{12}$$

where $c^* = c/c_0$. Substituting this expression into the divergence of the momentum equation - i.e., into $\nabla\cdot$ (10) - and only retaining terms of $\mathcal{O}\,(Ma)$ or larger yields

$$\frac{1}{(1+\alpha\Delta T_0\,\Delta T^*)\,{c^*}^2(\Delta T^*)}\left(\frac{\partial^2}{\partial {t^*}^2}+2Ma\,\boldsymbol{u}^*\cdot\nabla^*\frac{\partial}{\partial t^*}\right)p^*=\nabla^*\cdot\left(\frac{1}{1+\alpha\Delta T_0\,\Delta T^*}\nabla^*p^*\right). \tag{13}$$

As a final step, we note that the right hand side is explicitly written as

$$\frac{1}{1+\alpha\Delta T_0\,\Delta T^*}\nabla^{*2}p^* + \nabla^*\left(\frac{1}{1+\alpha\Delta T_0\,\Delta T^*}\right)p^* = \frac{1}{1+\alpha\Delta T_0\,\Delta T^*}\nabla^{*2}p^* + \mathcal{O}\left(\alpha\Delta T_0\frac{\lambda}{L}\right). \tag{14}$$

In our examplary scaling, we obtain

$$\alpha\Delta T_0\frac{\lambda}{L} \approx 10^{-5}, \tag{15}$$

which is smaller than the Mach Number and can therefore be neglected. We obtain the dimensionless form of the wave equation (17) in the main text

$$\frac{1}{c^{*2}(\Delta T^*)}\left(\frac{\partial^2}{\partial t^{*2}} + 2Ma\,\boldsymbol{u}^*\cdot\nabla^*\frac{\partial}{\partial t^*}\right)p^* - \nabla^{*2}p^* = 0. \tag{16}$$

Note that the scaling assumed for this derivation is in accordance with all flow and temperature models considered in our study. Some simplifications may not be appropriate for different dynamical regimes or fluid media.

## Derivation of the Adjoint Operator

Do derive the adjoint operator, we continue with in the dimensionless formulation adapted in the previous section. This makes identifying the relative scales between each term easier throughout the derivation. However, for convenience, we omit the star denoting a dimensionless variable and all quantities are dimensionless unless stated otherwise.

Testing the left hand side of the wave equation (Equation (16) in the previous section) with the adjoint wavefield $p^\dagger$ yields

$$\langle\mathcal{L}p, p^\dagger\rangle = \int_V\int_{[0,\mathcal{T}]}\left[\frac{1}{c^2(\Delta T)}\left(\frac{\partial^2}{\partial t^2} + 2Ma\,\boldsymbol{u}\cdot\nabla\frac{\partial}{\partial t}\right)p - \nabla^2 p\right]p^\dagger\,dV\,dt. \tag{17}$$

This Integral may be partitioned into three parts, such that

$$\langle\mathcal{L}p, p^\dagger\rangle = I_1 + I_2 - I_3, \tag{18}$$

where

$$I_1 = \int_V\int_{[0,\mathcal{T}]} p^\dagger\frac{1}{c^2(\Delta T)}\frac{\partial^2}{\partial t^2}p\,dV\,dt\,, \tag{19}$$

$$I_2 = \int_V\int_{[0,\mathcal{T}]} 2Ma\,p^\dagger\frac{1}{c^2(\Delta T)}\,\boldsymbol{u}\cdot\nabla\frac{\partial}{\partial t}p\,dV\,dt\,, \tag{20}$$

$$I_3 = \int_V\int_{[0,\mathcal{T}]} p^\dagger\nabla^2 p\,dV\,dt. \tag{21}$$

Applying integration by parts twice with respect to $t$ for $I_1$ results in

$$I_1 = \int_V\int_{[0,\mathcal{T}]} p\frac{1}{c^2(\Delta T)}\frac{\partial^2}{\partial t^2}p^\dagger\,dV\,dt + \int_V\left(p^\dagger\frac{1}{c^2(\Delta T)}\frac{\partial}{\partial t}p\bigg|_{t=0}^{t=\mathcal{T}} - p\frac{1}{c^2(\Delta T)}\frac{\partial}{\partial t}p^\dagger\bigg|_{t=0}^{t=\mathcal{T}}\right)dV \tag{22}$$

where all temporal derivatives of $c(\Delta T)$ are $\mathcal{O}\left(Ma\,\lambda/L\right)$ and can be neglected. This is equivalent to assuming frozen background fields while the sound wave is crossing the domain. The initial conditions $p(\boldsymbol{x},0) = \frac{\partial}{\partial t}p(\boldsymbol{x},0) = 0$ and terminal conditions $p^\dagger(\boldsymbol{x},\mathcal{T}) = \frac{\partial}{\partial t}p^\dagger(\boldsymbol{x},\mathcal{T}) = 0$ are now utilized to make the rightmost volume integral vanish. The same procedure can be applied for one temporal partial integration of $I_2$, which yields

$$I_2 = -\int_V\int_{[0,\mathcal{T}]} 2Ma\,\frac{1}{c^2(\Delta T)}\,\boldsymbol{u}\cdot\nabla p\,\frac{\partial}{\partial t}p^\dagger\,dV\,dt. \tag{23}$$

To move the spatial derivative onto the adjoint state, a Green's Identity may be used to obtain

$$I_2 = \int_V\int_{[0,\mathcal{T}]} 2Ma\,\frac{1}{c^2(\Delta T)}\,p\,\boldsymbol{u}\cdot\nabla\frac{\partial}{\partial t}p^\dagger\,dV\,dt - \int_{\partial V}\int_{[0,\mathcal{T}]} 2Ma\,\frac{1}{c^2(\Delta T)}\hat{\boldsymbol{n}}\cdot\boldsymbol{u}\,p\,\frac{\partial}{\partial t}p^\dagger\,dS\,dt, \tag{24}$$

where $\int_{\partial V} dS$ denotes the integral along the boundary $\partial V$ of the domain. Here, the spatial derivative of $c(\Delta T)$ has been neglected, as the resulting term is of magnitude $\mathcal{O}\left(Ma\,\lambda/L\right)$. Furthermore, the surface integral in the right term vanishes since the flow must obey a non-penetration condition $\hat{\boldsymbol{n}} \cdot \boldsymbol{v} = 0$ at the boundary $\partial V$ of the domain. For the third integral, another one of Green's Identities (i.e. Gauss' Theorem) may be used to write

$$I_3 = -\int_V \int_{[0,\mathcal{T}]} \nabla p^\dagger \cdot \nabla p \, dV \, dt + \int_{\partial V} \int_{[0,\mathcal{T}]} p^\dagger \left(\hat{\boldsymbol{n}} \cdot \nabla p\right) \, dS \, dt. \tag{25}$$

Using the Neumann boundary condition for the pressure, the boundary integral also vanishes.
A last manipulation leads to

$$I_3 = \int_V \int_{[0,\mathcal{T}]} p \, \nabla^2 p^\dagger \, dV \, dt - \int_{\partial V} \int_{[0,\mathcal{T}]} p \left(\hat{\boldsymbol{n}} \cdot \nabla p^\dagger\right) \, dS \, dt \,, \tag{26}$$

where also a Neumann condition $\hat{\boldsymbol{n}} \cdot \nabla p^\dagger = 0$ has to be imposed for the adjoint wavefield on $\partial V$ to make the boundary integral vanish.
Consequently, all three integrals can be recombined to yield

$$\langle \mathcal{L}p, p^\dagger \rangle = \int_V \int_{[0,\mathcal{T}]} p \left[ \frac{1}{c^2(\Delta T)} \left( \frac{\partial^2}{\partial t^2} + 2Ma\,\boldsymbol{u} \cdot \nabla \frac{\partial}{\partial t} \right) p^\dagger - \nabla^2 p^\dagger \right] dV \, dt \, = \langle p, \mathcal{L}^\dagger p^\dagger \rangle \,, \tag{27}$$

which makes it clear to see that the forward operator $\mathcal{L}$ and the adjoint operator $\mathcal{L}^\dagger$ are indeed the same. That is, the operator $\mathcal{L}$ is self-adjoint.
